# Bibliographical Notices

**Published:** 1844-02-10

**Authors:** 


### BIBLIOGRAPHICAL NOTICES.

*On Inflammation and Abscess of the Uterine Appendages.* By Fleetwood Churchill, M. D., M. R. I. A. &c. (Read before the Surgical Society (of Dublin), January 28th, 1843.)

The subject of this Essay is "inflammation of the uterine appendages, terminating most frequently in abscess, sometimes unconnected with childbirth, but more frequently following delivery within a longer or shorter time."

In the opinion of the author, "the affection is sufficiently frequent to merit our careful attention, and there is reason to believe that it is even more so than has been suspected; nor is it improbable that many females have owed the delicate health in which they have remained for some time after confinement, to this condition of the parts, undetected because of the obscurity of the symptoms; neither has it been very distinctly recognized by writers on the diseases of the womb,—their observations are slight and the cases few." The histories of twenty-three cases are given, occurring under the observation of the author, or extracted from the writings of Clarke, Puzos, and others, illustrating the subject of the paper. "Inflammation of the uterine appendages may occur, he remarks, in an acute or chronic form. In the former, it constitutes one of the varieties of puerperal fever, and has been most ably treated by Clarke, Lee, Ferguson, Puzos, &c." Dr. Churchill therefore confines his remarks chiefly to the disease in the chronic form. The causes, mode of invasion, symptoms, terminations, diagnosis, and treatment, are discussed in the peculiarly terse and forcible manner so characteristic of all the author's productions.

*The Anatomy and Surgical Treatment of Abdominal Hernia. With numerous plates.* By Sir Astley Cooper, Bart., F. R. S., Surgeon to the King, and Consulting Surgeon to Guy's Hospital. *From the Second London Edition.* By C. Aston Key, Senior Surgeon to Guy's Hospital, and Lecturer on Surgery. Imperial octavo, pp. 427. Philadelphia: Lea and Blanchard, 1844.

This elaborate work of the English Baronet, is not now before us for the first time, to challenge criticism or excite remark. Forty years have elapsed since it was originally published in London, and the experience of that long period has confirmed the accuracy of its delineations, and the excellence of its precepts. Hitherto, however, its costliness and inconvenient size have kept it out of general circulation; and we rejoice that the Philadelphia publishers have brought it out in a form and at a price that will render it accessible to all.

To such as have never seen the work, we will observe that it contains a full and accurate description of "The Anatomy and Surgical Treatment of Abdominal Hernia."

It has been a primary object with the author "to trace the progress of the disease from its commencement, to explain the nature and uses of the parts contiguous to hernia, which are in any way connected with its formation and increase; and especially to describe, with sufficient minuteness, the surrounding arteries, as far as their course interferes with the operations of the surgeon;" and, likewise, "to lay down rules for operating, calculated to meet all the varieties of Hernia hitherto discovered." In the attainment of these objects the distinguished author has been eminently successful, and the work is unquestionably one of the most valuable practical treatises on the subjects on which it is written that have ever been presented to the profession.

The present edition contains numerous plates, lithographed by Sinclair, in his very superior manner. It is printed on strong white paper, with new type, and is altogether extremely creditable to the American press.

